# Picosecond spin-orbit torque–induced coherent magnetization switching in a ferromagnet

**DOI:** 10.1126/sciadv.adh5562

**Published:** 2023-09-06

**Authors:** Debanjan Polley, Akshay Pattabi, Ashwin Rastogi, Kaushalya Jhuria, Eva Diaz, Hanuman Singh, Aristide Lemaitre, Michel Hehn, Jon Gorchon, Jeffrey Bokor

**Affiliations:** ^1^ Lawrence Berkeley National Laboratory, 1 Cyclotron Road, Berkeley, CA 94720, USA.; ^2^ Department of Electrical Engineering and Computer Sciences, University of California, Berkeley, CA 94720, USA.; ^3^ Department of Physics and Nanotechnology, SRM Institute of Science and Technology, Kattankulathur- 603 203, Tamil Nadu, India.; ^4^ Department of Engineering, University of San Francisco, San Francisco CA 94117, USA.; ^5^ Université de Lorraine, CNRS, IJL, Nancy, France.; ^6^ Université Paris-Saclay, CNRS, Centre de Nanosciences et de Nanotechnologies, 91120, Palaiseau, France.

## Abstract

Electrically controllable nonvolatile magnetic memories show great potential for the replacement of conventional semiconductor-based memory technologies. Here, we experimentally demonstrate ultrafast spin-orbit torque (SOT)–induced coherent magnetization switching dynamics in a ferromagnet. We use an ultrafast photoconducting switch and a coplanar strip line to generate and guide a ~9-picosecond electrical pulse into a heavy metal/ferromagnet multilayer to induce ultrafast SOT. We then use magneto-optical probing to investigate the magnetization dynamics with sub-picosecond resolution. Ultrafast heating by the approximately 9 picosecond current pulse induces a thermal anisotropy torque which, in combination with the damping-like torque, coherently rotates the magnetization to obtain zero-crossing of magnetization in ~70 picoseconds. A macro-magnetic simulation coupled with an ultrafast heating model agrees well with the experiment and suggests coherent magnetization switching without any incubation delay on an unprecedented time scale. Our work proposes a unique magnetization switching mechanism toward markedly increasing the writing speed of SOT magnetic random-access memory devices.

## Teaser

Ultrafast spin-orbit-torque drives coherent magnetization switching in a ferromagnet within ~70 ps due to ~9 ps current pulse.

## INTRODUCTION

In recent years, spintronics (*1*–*3*) has gained considerable attention and shows great promise for low-power, nonvolatile memory technology. While magnetic memory devices based on spin-transfer torque (STT) have already been introduced to the market (*4*–*6*), they still present some challenges such as device lifetime (10^10^ write cycles limited by tunnel barrier breakdown), relatively slow switching speed and the write error rate (due to the stochastic thermal fluctuation induced switching initiation), which are affecting its performance compared to the state-of-the-art semiconductor memory devices (*3*, *4*). Spin-orbit torque (SOT)–based devices, on the other hand, are expected to largely overcome these problems. Current-induced SOT switching possesses notable advantages over current-induced STT switching as the former does not require the switching current to pass through the MgO tunnel barrier. In STT devices with perpendicular magnetic anisotropy (PMA) (widely used due to favorable dimensional scaling properties), the torque is parallel to the magnetization of the free layer and requires thermal fluctuations to initiate the switching process leading to a stochastic incubation delay (*7*, *8*). On the other hand, SOT is orthogonal to the magnetization of the free layer, which is expected to provide “instant-on” switching torque. However, recent studies of SOT switching dynamics on the approximately nanosecond time scale in ferromagnet/ferrimagnet also reveal an incubation delay (*9*), which is attributed to the stochastic nature of the domain wall nucleation (*8*–*10*). In most experiments, the operation speed of SOT devices, i.e., the observed switching time scale for the magnetic layer, has been limited by the rise time and width of the current pulse source (typically in the approximately nanosecond range) (*8*, *11*–*14*). For comparison, a ferrimagnet (*15*, *16*) or hybrid ferrimagnet/ferromagnet structures (*17*–*20*) can be switched in a few picoseconds using the thermally activated helicity-independent all-optical switching mechanism. This switching can also be manifested using approximately picosecond current pulses obtained from appropriately designed, optically excited ultrafast photoconductive (Auston) switches (*21*). Coherent reversal of in-plane magnetic dots in ~200 ps has been previously reported using a specially designed magnetic field pulse generator from two coupled Auston switches (*22*). Recently, SOT-induced switching in a ferromagnet (*23*) with PMA has been demonstrated using ~6-ps current pulses from an Auston switch embedded in a coplanar waveguide. A temperature-dependent Landau-Lifshitz-Gilbert (LLG)–based macro-magnetic simulation (*23*) suggested a coherent rotation of magnetization with the switching time scale being an order of magnitude faster than conventional approximately nanosecond SOT switching events which are governed by domain wall nucleation and propagation. However, experimental measurements of the switching dynamics were not reported in that work.

Here, we generate ~9-ps current pulses from an Auston switch embedded in a coplanar strip line (CPS) waveguide, guide them into a magnetic heterostructure containing heavy metal and Co ferromagnetic layers, and study the resulting ultrafast SOT-induced switching dynamics. Time-resolved magneto-optical Kerr effect (MOKE) experiments allow us to observe the ultrafast magnetization dynamics as a function of the current pulse and in-plane symmetry-breaking magnetic field (*H_x_*) direction and amplitude. We observe ultrafast magnetization switching (zero crossing) in ~70 ps after the approximately picosecond current pulse excitation, even for our device size of ~5 × 4 μm^2^, which is an order of magnitude faster compared to previous SOT switching studies using much smaller (approximately hundreds of nanometer) device dimensions (*8*, *10*, *13*). Full reversal is achieved in ~250 ps, set by the time for the ferromagnetic layer to cool via heat diffusion to the substrate. The switching speed cannot be understood in terms of current-driven domain wall dynamics. We use a modified LLG equation-based macrospin simulation, with SOT-induced torques, including ultrafast thermal heating effects to explain the experimentally measured time-resolved magnetization dynamics, which provides important insight into the mechanism of the switching phenomena. The ultrafast switching is attributed to the coherent rotation of magnetization by a large damping-like torque (τ_DL_) and ultrafast thermal anisotropy torque (τ_TAT_) of the softened magnet due to ultrafast joule heating by the approximately picosecond current pulse.

## RESULTS

**Fig. 1.**
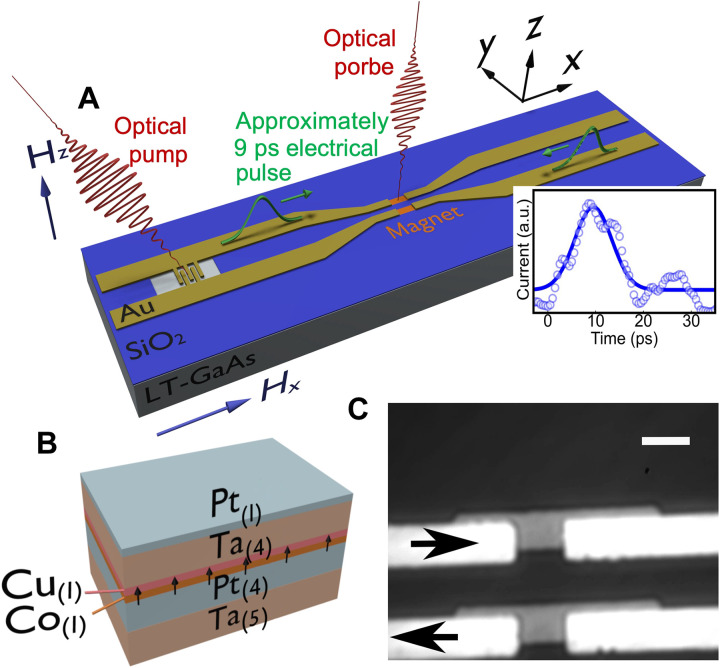
Schematic of measurement technique and sample structure. (**A**) Schematic diagram of the Auston switch embedded in the CPS along with the optical pump and probe laser pulse and the approximately picosecond current pulse propagation, while the bottom right inset shows measured current pulse (blue circles) using the Protemics tip and a Gaussian fitting (blue line) gives us ~9-ps full width at half maximum. (**B**) Schematic of the cross-sectional view of the magnetic multilayer where the black arrows denote PMA in the 1-nm Co layer and (**C**) the optical image of the magnet embedded in the CPS structure. The white rectangular slabs are the metallic electrodes of the CPS and the slightly off-center darker rectangular slabs are the magnetic multilayer. The black arrows designate the approximately picosecond current pulse propagation direction for a positive bias across the Auston switch. The white scale bar is ~5 μm.

We used a magnetic multilayer structure containing a 1-nm-thick Co film with PMA grown on top of a low-temperature grown GaAs substrate and patterned into magnetic device regions with dimensions of ~5 × 4 μm^2^. We then fabricated a CPS microwave waveguide (as shown in Fig. 1A) with the magnetic stack embedded in both conductor lines. The ferromagnet has a coercivity of ~200 Oe (see section S1) and a multilayer stack structure Ta_(5)_/Pt_(4)_/Co_(1)_/Cu_(1)_/Ta_(4)_/Pt_(1)_, where the thickness of each layer is given in nanometers in the subscript and schematically shown in Fig. 1B. Details about the sample fabrication can be found in Materials and Methods and elsewhere (*21*, *23*). The amplitude and the temporal shape of the current pulse were measured using a terahertz microprobe from Protemics GmbH, and we observe a Gaussian-like current pulse of ~9-ps full width at half maximum (FWHM) as shown in the top inset of Fig. 1. Details of the electrical characterization of the Auston switch are given in section S2 and are similar to previous reports (*21*, *23*). The differential MOKE images of the magnetic microdots after exciting them with single-shot electrical pulses in the presence of 1600 Oe in-plane symmetry-breaking magnetic field (*H_x_*) are shown in section S3. These images indicate that the final magnetic states of the microdots are independent of the initial states and depend only on the relative orientation of the approximately picosecond current pulse and *H_x_* as expected from the symmetries of the SOT in the magnetic heterostructures (*13*, *23*).

**Fig. 2.**
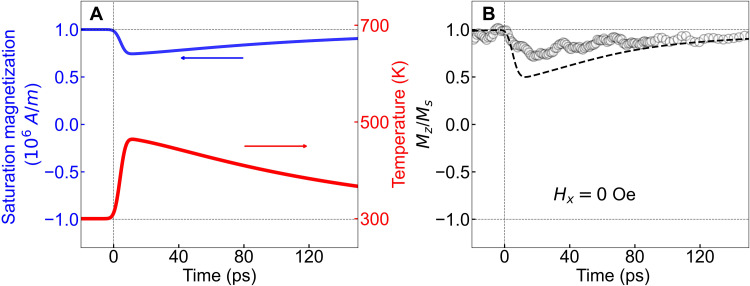
Ultrafast magnetization dynamics without any in-plane symmetry-breaking magnetic field. (**A**) Evolution of temperature (red) as a function of time due to the 9-ps current pulse as calculated from the one-temperature model and the corresponding ultrafast change in saturation magnetization (blue) due to thermal heating by the approximately picosecond current pulse (does not include any SOT effect). (**B**) The experimentally measured time-resolved ultrafast magnetization dynamics (in the absence of any in-plane magnetic field) induced by the approximately picosecond current pulse excitation are shown in black circles. The dashed black line shows the simulation considering a macroscopic LLG model which includes ultrafast SOT effects, with Θ_DL_ = 0.2; Θ_FL_ = 0.04, and ultrafast thermal effects (as calculated in Fig. 2A) due to the 9-ps current pulse with a current density of 7.3 × 10^12^ A/m^2^.

**Fig. 3.**
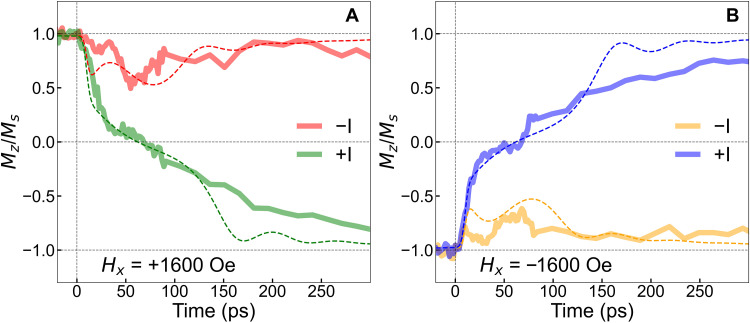
Ultrafast current pulse–induced magnetization switching dynamics. The approximately picosecond current pulse–induced time-resolved magnetization dynamics measured in the presence of (**A**) positive and (**B**) negative in-plane symmetry-breaking magnetic field and reversing the direction of the current pulses starting from positive and negative magnetic saturation. The dotted lines show the theoretical analysis by solving a macroscopic LLG equation with damping-like (Θ_DL_ = 0.2) and field-like (Θ_FL_ = 0.04) SOT terms and combining ultrafast one-temperature heating model due to a 9-ps current pulse with a current density of ±7.3 × 10^12^ A/m^2^.

We measured the ultrafast magnetization dynamics using a conventional time-resolved pump-probe polar MOKE setup (*19*, *21*). The details of the experimental setup can be found in Materials and Methods. We apply a ~3.7-mJ/cm^2^ incident optical fluence (laser pulse) on the Auston switch with a bias voltage of +50 V which drives the Auston switch to full saturation to generate the current pulse with ~9-ps FWHM as shown in the inset of Fig. 1. Increasing the bias voltage beyond 50 V together with the laser pulse irradiation caused damage to the Auston switch. The ~9-ps current pulse with a high current density introduces noticeable ultrafast joule heating into the magnetic multilayer system. The electrons and phonons can safely be estimated to be in thermal equilibrium during the entire duration of the ~9-ps current pulse. Hence, we have calculated the ultrafast thermal change via a one-temperature (1-T) model. The evolution of temperature and the corresponding change in the saturation magnetization $\left[M_{s}(T)=M_{s}(0K){\left(1-T_{C}/T\right)}^{1.7}\right]$ is plotted in Fig. 2A via the red and blue lines, respectively. It is worth mentioning that in this 1-T model, we do not consider the multilayer nature of the sample structure, external magnetic fields, and the temperature-dependent anisotropy. We believe that these present some limitations in capturing the true nature of the complex heat distribution and saturation magnetization evolution. We obtain an ultrafast temperature rise to ~460 K and an associated ultrafast thermally induced demagnetization close to ~24%. This effect of ultrafast heating is present in all the subsequent measurements and has been incorporated in the modified LLG-based macro-magnetic simulation of the magnetization dynamics. The details of the LLG simulation can be found in section S4. The ~9-ps current pulse–induced magnetization dynamics in the absence of *H_x_* (*H_z_* = 300 Oe; *H_x_* = 0 Oe) are shown in Fig. 2B in black circles. Both the LLG simulation and the polar MOKE experiment calculate the normalized (with respect to saturation magnetization at 300 K) out-of-plane component of the magnetization, which is henceforth denoted as $M_{z}/M_{s}$. Experimentally, we observe a ~30% change in $M_{z}/M_{s}$ within the first 20 ps of the electrical excitation. The magnetization dynamics in the ferromagnetic microdot (as shown by black open circles in Fig. 2B) partly arise from ultrafast heating induced by the approximately picosecond current pulses (as calculated in Fig. 2A). The dynamics are further driven by the ultrafast SOT (even in the absence of *H_x_*) generated by the propagation of the approximately picosecond current pulse through the heavy-metal layer in the magnetic heterostructure. The measured magnetization drop in $M_{z}/M_{s}$ at the shorter time scale and its recovery at a longer time scale in Fig. 2B is slightly different compared to the simulated evolution of the saturation magnetization as shown in Fig. 2A due to the reasons discussed before. The simulated dynamics (including the ultrafast heating effect) in the absence of *H_x_* (*H_z_* = 300 Oe; *H_x_* = 0 Oe) are shown by the dashed black line in Fig. 2B considering damping-like and field-like spin Hall angles of Θ_DL_ = 0.2 and Θ_FL_ = 0.04, respectively, for a 9-ps Gaussian current pulse with a current density of +7.3 × 10^12^ A/m^2^ (the current density has been fixed by fitting the experimental data shown in Fig. 3 and will be discussed later). A separate measurement of the ultrafast demagnetization of the magnetic microdot due to direct optical excitation by a ~100-fs laser pulse is shown in section S5, where we have observed ~50% magnetization change due to an absorbed laser fluence of ~0.3 mJ/cm^2^.

We observed the ultrafast SOT-induced magnetization switching dynamics via time-resolved MOKE starting from both a positive and negative magnetic saturation state, and for different in-plane field and current directions, as shown in Fig. 3 (A and B). The simulated magnetization dynamics using the LLG-based macrospin simulation (including the ultrafast heating effect) are shown by the dashed lines in Fig. 3 using Θ_DL_ = 0.2 and Θ_FL_ = 0.04, for a 9-ps Gaussian current pulse with a current density of ±7.3 × 10^12^ A/m^2^ in the presence of a ±1600-Oe *H_x_* and 300-Oe out-of-plane bias magnetic field (*H_z_*). Unless otherwise specified, the amplitude of *H_x_* is always 1600 Oe (directions vary) in the subsequent experimental and simulated results.

We observe reasonable agreement of the simulated dynamics with the experimental data. Starting from positive magnetic saturation in the presence of a positive *H_x_*, we notice a modified initial drop in the out-of-plane magnetic component relative to the zero in-plane field case (which is discussed earlier in Fig. 2B) due to a negative approximately picosecond current pulse as shown in Fig. 3A in a solid red line. The corresponding remagnetization at a longer time scale is accompanied by precessional dynamics with two prominent magnetization oscillations (one smaller oscillation peak at ~30 ps and another larger oscillation peak at ~60 ps). Similar dynamics are observed starting from a negative magnetic saturation in the presence of a negative *H_x_*, as shown by the solid orange line in Fig. 3B. The effect of the positive approximately picosecond current pulse, starting from positive magnetic saturation in the presence of a positive *H_x_*, is shown in solid green lines in Fig. 3A. Here, we detect a zero crossing in ~70 ps and a nearly complete switching [magnetization reaches ~75% in the opposite direction (*24*)] in ~250 ps. Again, similar dynamics are observed starting from a negative magnetic saturation in the presence of a negative *H_x_*, as shown by the solid blue line in Fig. 3B. We notice that the magnetization does not recover to its full reversed saturation value after switching even after 600 ps, as shown in the long time-delay scans (section S6). This is attributed to the time required for the device to fully recover to room temperature via heat diffusion into the substrate in our sample. We show the ultrafast SOT-driven magnetization dynamics for a positive (and negative) approximately picosecond current with increasing optical pump energy on the Auston switch in the presence of a constant *H_x_* in section S7. The zero-crossing time and the overall shape of the dynamics do not change with the increasing optical fluence on the Auston switch as it is already operating at saturation at 0.28-μJ optical energy (~3.7-mJ/cm^2^ optical fluence). Ultrafast thermal heating by approximately picosecond current pulse and the subsequent demagnetization and thermal anisotropy reduces the required current density of magnetization switching (when compared to the current needed to switch the magnetization without any ultrafast heating) as described in section S2.

**Fig. 4.**
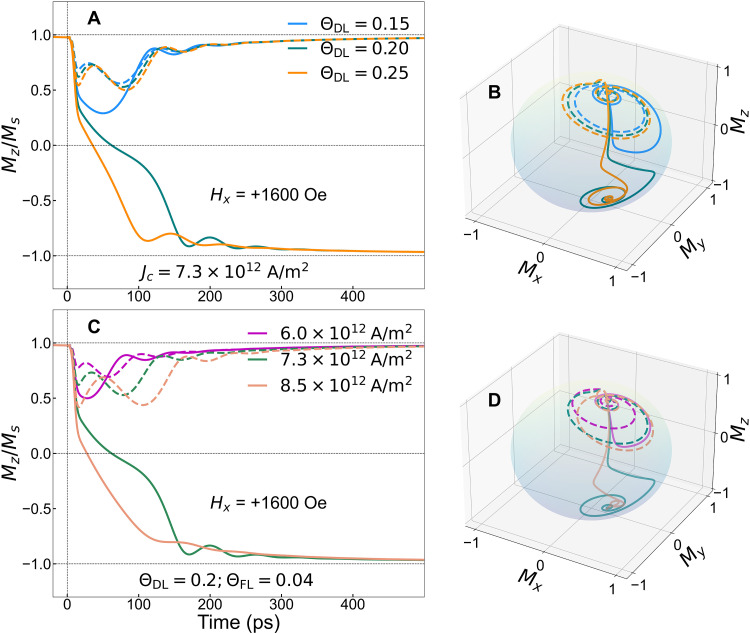
Simulated effects of ultrafast current-driven torques on the magnetization dynamics. The simulated magnetization dynamics using a modified LLG-based macro-magnetic model (including the ultrafast heating effect) (**A**) for different values of damping-like (Θ_DL_) torque (at a fixed field-like torque of Θ_FL_ = 0.04) using a 9-ps current pulse with a fixed current density of 7.3 × 10^12^ A/m^2^ and (**B**) the corresponding three-dimensional dynamics of the magnetization vector for three different damping-like torque values. (**C**) The magnetization dynamics for different current densities at a fixed value of Θ_DL_ = 0.2 and Θ_FL_ = 0.04, in the presence of a 1600 Oe symmetry-breaking in-plane field and the (**D**) corresponding three-dimensional dynamics of the magnetization vector. The solid (dotted) lines show the magnetization dynamics for positive (negative) current pulses.

The theoretical analysis for the effect of different values of Θ_DL_ (at a fixed Θ_FL_ = 0.04) on the ultrafast magnetization dynamics is shown in Fig. 4A using the 9-ps current pulse with a current density of 7.3 × 10^12^ A/m^2^, and the corresponding three-dimensional magnetization trajectories are shown in Fig. 4B. We observe magnetization switching (with positive current pulses) for a larger Θ_DL_, and the zero-crossing times become faster with increasing Θ_DL_. For negative current pulses (as shown by the dotted lines in Fig. 4, A and B), we do not observe magnetization switching. However, τ_DL_ tilts the magnetization in the opposite direction (compared to τ_TAT_), which is observed by the initial change in the magnetization, and the magnetization oscillation amplitude increases with increasing τ_DL_. The effect of different current densities at a fixed value of Θ_DL_ = 0.2 and Θ_FL_ = 0.04 is shown in Fig. 4C along with the three-dimensional magnetization trajectories shown in Fig. 4D. Magnetization dynamics at relatively low current densities are accompanied with either slower or no-switching. With increasing current density, the effect of the thermal anisotropy torque becomes larger as the ultrafast heating gets stronger. The ultrafast SOT effects by the approximately picosecond current pulse also increase due to the increasing current density, which introduces more tilting of the magnetization within the current pulse duration due to increased τ_DL_. As a combined effect, we observe faster zero crossing for positive approximately picosecond current pulses with increasing amplitude.

## DISCUSSION

The thermal heating by the approximately picosecond current pulse creates a time-dependent change not only in the magnetization amplitude but also in the anisotropy field (for details, see section S4). The ultrafast demagnetization reduces the magnetization amplitude and τ_DL_ tilts the magnetization. The tilted magnetization and the effective anisotropy field (time-dependent anisotropy field in combination with a constant external *H_x_* and *H_z_*) become non-colinear and create a torque that lasts much longer than the current pulse duration. This is defined as τ_TAT_, whose effect depends both on *H_x_* and ultrafast thermal demagnetization.

A negative current pulse does not induce switching (for the chosen combinations of initial magnetization saturation and in-plane magnetic field directions in Fig. 3) but generates ultrafast demagnetization together with SOT-induced oscillations, whose amplitude and period are governed by the magnitude of the damping torque and the temporal evolution of the effective anisotropy field. The effect of τ_DL_ tilts the magnetization in the opposite direction (compared to τ_TAT_), and τ_TAT_ initiates strong magnetization oscillations. As the anisotropy field (*H*_ani_) is a function of temperature (which is again a function of time), the frequency of oscillation changes proportional to $2\pi /{\gamma H}_{\mathrm{ani}}$, which is responsible for the two prominent oscillation peaks centered at ~30 and ~60 ps as shown in red (and orange) solid lines in Fig. 3A (see also Fig. 3B). The effect of the τ_DL_ vanishes after the finite duration of the current pulse (~25 ps), and the subsequent magnetization dynamics are then driven by τ_TAT_. The oscillations get damped within ~250 ps due to the large Gilbert damping of the material (*23*), and it aligns the magnetization along the effective magnetic field at a longer time scale due to the Gilbert damping torque (τ_GL_). We notice that the dynamics are not perfectly symmetric when starting from positive and negative saturations. This can be due to asymmetries in the ultrafast torques coming from (i) anisotropic current pulses in the CPS lines or/and (ii) a difference in the effective in-plane magnetic field between positive and negative orientation (due to nonidealities in the external magnet structure used to apply the in-plane and out-of-plane magnetic fields).

The effect of *H_x_* on the ultrafast magnetization dynamics is shown in section S8, where we observe that an 800-Oe in-plane symmetry-breaking field is insufficient to observe magnetization switching with the same current density. For the positive approximately picosecond current pulse, τ_TAT_ and τ_DL_ work in tandem and try to drag the magnetization away from its equilibrium. However, with the smaller in-plane field, the magnitude of τ_TAT_ is smaller. Hence, to induce complete magnetization switching, a larger current density or *H_x_* is required.

The FWHM of the current pulse obtained from the Auston switch can vary between ~6 to 10 ps due to electrode geometry (*21*, *23*). Reliable ultrafast SOT-induced switching with ~6-ps current pulses has previously been reported by Jhuria *et al*. (*23*) although the time-resolved switching dynamics were not explored. In the macroscopic simulation, we varied the current pulse width between 6 and 15 ps and observed consistent SOT-induced switching within ~100 ps. The switching mechanism and the underlying physics remain the same within the range of the simulated current pulse width. However, the required current density reduces considerably with increasing FWHM as shown in section S2. This would be important in a memory device application in terms of smaller device size where the smaller peak current density can be obtained from a smaller transistor.

### Ultrafast magnetization switching mechanism

The physics of SOT at the sub-nanosecond time scale is a matter of intense debate. The major issue involves the seemingly different switching mechanisms at the approximately nanosecond and approximately picosecond time scales. Most of the previously reported SOT-induced switching experiments were performed with approximately nanosecond current pulses where the switching dynamics are governed by domain wall nucleation and propagation (*8*–*10*, *12*, *14*, *25*). The typical SOT-induced domain wall velocities are in the range of 0.1 to 0.5 km/s in the commonly used ferromagnets like Co or CoFeB (*8*, *13*, *14*) but can reach up to ~5 km/s in Gd-based ferrimagnets near compensation or in special ferromagnets with ultrasmall Gilbert damping (*12*, *26*) as observed in earlier studies. In contrast, the reversal mechanism at the ps time scale can possibly involve a coherent rotation of the magnetization. Considering a ~250-ps switching time, we infer an unrealistic domain wall velocity of ~25 km/s [exceeding the Walker breakdown (*26*)] in our Co microdot with ~5 μm by 4 μm dimensions. Hence, we conclude that the underlying mechanism must be coherent (and not domain wall–driven) to be so fast. Furthermore, classical domain wall nucleation is generally considered to be a stochastic process with an average nucleation time of the order of 1 ns or greater (*8*, *9*, *14*, *25*). We observe essentially no incubation time in our experiments. We do not resolve single-shot dynamics, as reported in some recent studies (*8*, *9*). However, a random incubation delay of approximately nanosecond duration would manifest as a stretched-out rise time of the averaged repetitive pump-probe signal, which we do not observe in our experiment. It has been previously envisioned that the incubation time to nucleate a domain wall can be almost completely suppressed by a strong perturbation with shorter current pulses, which is called an intrinsic SOT switching regime (*9*, *13*). Simulations show that in this regime, SOT itself is sufficient to take the magnetization away from the equilibrium position to nucleate a domain wall without the need for any thermal energy (thermal rise time, ~2 ns) (*9*). However, our measurement scenario is completely different as we use a approximately picosecond current pulse and it induces a strong and ultrafast thermal change responsible for the demagnetization occurring in tens of ps as observed in Fig. 2A. The magnetization responds almost instantaneously to the applied approximately picosecond current pulse with no incubation delay. These observations suggest that we are operating at a nearly coherent regime with the intense approximately picosecond current pulse, which is again supported by the macro-magnetic simulation. There could conceivably be simultaneous instantaneous nucleation of numerous closely spaced small domains across the device, which then expand to then merge and switch the device on such a fast time scale. However, to our understanding, essentially such numerous multiple domain nucleation should cover more than 50% of the device size (*23*), which would equivalently indicate virtually coherent switching of the magnetization across the full area of the structure.

When we apply a positive current pulse starting from positive saturation in the presence of positive *H_x_*, the current pulse partially demagnetizes the film on an ultrafast time scale (*21*, *23*), and, at the same time, τ_DL_ tilts the magnetization along the spin-polarization axis. The τ_TAT_ initiates strong magnetization oscillations. In this case, the τ_DL_ acts opposite to that of the negative current pulse direction. Hence, the effect of τ_DL_ gets enhanced by τ_TAT_. However, damping-like torque vanishes after the current pulse drops essentially to zero (~25 ps). Then, the magnetization continues to be driven by τ_TAT_ and it crosses the equatorial plane. At a longer time scale, τ_GL_ relaxes the magnetization along the opposite saturated direction (*27*). Similar dynamics are observed for a positive current pulse starting from negative magnetic saturation, using a negative *H_x_*. This switching mechanism discussed here has been hypothesized in an earlier work (*23*) and these measurements now corroborate the hypothesis. We highlight that this precessional switching mechanism can only work if the heating, and change in effective field direction, happen at a faster time scale than the ferromagnetic resonance half-period. Otherwise, for slower heating, the magnetization would simply follow the effective field and could not swing across the equator plane unless the effective field itself did cross it. Our model mimics the zero-crossing time of ~70 ps and shows close agreement with the shape of the experimental magnetization switching curve as shown in Fig. 3. In the simulated switching dynamics, the magnetization gets settled to its complete switched value faster than the experiment. In the simulation, we have not considered the effect of the oxide layer in between the magnetic layer and substrate, which might slow cooling back to ambient temperature to cause such a discrepancy. The ultrafast thermal heating, which is required to obtain such fast magnetization switching, could be problematic in some practical applications. Engineering smaller magnetic element sizes with better thermal transport properties could lead to an optimized solution.

The effect of the damping and the thermal anisotropy torques works in the same direction for positive and in the opposite direction for negative current pulses. With a positive current pulse (shown in the solid lines in Fig. 4, A and B), for the smallest damping-like SOT term (Θ_DL_ = 0.15), the out-of-plane magnetization tilts sharply; however, together with τ_TAT_, it is still insufficient to produce enough torque for the magnetization to cross the equator and, ultimately, it settles along its initial direction. Now, for negative current pulses, the τ_TAT_ working in the opposite direction initiates the oscillations and we observe increased oscillation radius with increasing τ_DL_ from the dotted lines in Fig. 4B. The oscillations get damped by the strong τ_GL_ at longer time scales. The effect of the larger τ_TAT_ is also reflected in the increased oscillation amplitudes and reduced frequency $\left(\propto 2\pi /{\gamma H}_{\mathrm{ani}}\right)$ along the negative current direction before the magnetization settles toward its initial saturation. Ultrafast demagnetization, and the effect of τ_DL_ and τ_TAT_, increases with an increasing current density of the approximately picosecond electrical pulse (at a fixed Θ_DL_ and Θ_FL_). Consequently, we detect a much larger amplitude magnetization oscillation during remagnetization (for negative current direction) and a much faster magnetization zero crossing (for positive current pulse direction). The value of Θ_DL_ and Θ_FL_ depends on the heavy-metal layer and the ferromagnet/heavy-metal interface. For our magnetic stack, the effect of τ_FL_ is minimal as observed in the previous studies (*23*, *28*) and also corroborated by our simulation (see section S9). After zero crossing, the magnetization stabilization gets delayed with increasing field-like torque. The coherent nature of the observed ultrafast magnetization reversal is well established with our macrospin model, and it suggests the possibility of achieving an even faster (~20 ps) zero-crossing time.

### Energy density

Our simulated current density (*J_c_* = 7.3 × 10^12^ A/m^2^) from the macroscopic model is an order of magnitude larger when compared to approximately nanosecond current pulse–induced SOT switching measurements (*9*, *29*). However, this current density is needed only for ~9 ps (instead of up to tens of nanoseconds), and hence, the corresponding maximum energy density [~0.75 × $J_{c}^{2}$(*t*)ρτ] is ~0.30 aJ/nm^3^ is small. The required energy would reduce only to ~3 fJ in an actual device with a 20 nm by 20 nm by 20 nm size magnetic dot, which is notably smaller than the reported switching energy values when using approximately nanosecond current pulses (*12*). The driver transistor size limits the final SOT–magnetoresistive random access memory (MRAM) cell size as a larger current can only be delivered by a larger transistor, which will ultimately increase the array size or reduce the memory density. Hence, substantial improvement is required to obtain faster switching at an optimal current density and we believe heavy-metal layers or topological insulators with much larger SOT efficiency can be an important step forward to reduce the required current density. SOT efficiency can also be larger than 1 (*30*, *31*) in specially designed current-carrying materials, which promises an even smaller threshold current density in an optimized magnetic stack. Some recent studies reveal that the use of a small STT current in addition to a SOT current can reduce the switching current density (*32*). The ultrashort current pulse width may also help in enhancing the device lifetime, which can be damaged by electromigration and self-heating induced diffusion, as has been observed in recent theoretical works comparing 100- and 1-ns current pulses (*33*).

In summary, we have demonstrated time-resolved ultrafast SOT-induced magnetization dynamics in a 1-nm Co film using ~9-ps current pulses generated from an optically excited voltage-biased photoconducting Auston switch. The current pulse induces ~24% ultrafast thermal demagnetization in the ferromagnetic microdot due to ultrafast thermal heating. The time-resolved magnetization dynamics reveal a magnetization zero crossing at ~70 ps, while the full switching takes about ~250 ps where with the full recovery of the switched magnetization amplitude limited by heat diffusion from the ferromagnetic microdot to the substrate. A macrospin LLG simulation (containing a dominant damping-like and a small field-like torque) combined with ultrafast thermal response agrees well with the experimentally observed magnetization dynamics. While previous SOT experiments with approximately nanosecond long current pulses confirm domain wall nucleation and propagation-driven magnetization dynamics, our present measurement with ultrafast SOT shows a magnetization reversal by a rapid coherent rotation of the partially demagnetized magnetic moments toward the switched direction. We hypothesize that this difference may be attributed to a combination of strong peak driving torque combined with the transient ultrafast heating and thermal anisotropy torque, which overtakes the time scale for domain wall nucleation. This work points the way toward achieving integrated, on-chip SOT switching on sub-100-ps time scales. Our work sets an important milestone in demonstrating the possibility of coherent ultrafast SOT switching and offers a unique realm of ultrafast magnetism, combining nonequilibrium heating with SOT effects.

## MATERIALS AND METHODS

### Sample fabrication

We used dc magnetron sputtering to grow a magnetic multilayer structure containing a 1-nm Co film with PMA on top of a low-temperature grown GaAs layer on a GaAs bulk substrate and patterned magnetic device regions with dimensions of ~5 μm by 4 μm. The multilayer stack structure is Ta_(5)_/Pt_(4)_/Co_(1)_/Cu_(1)_/Ta_(4)_/Pt_(1)_, where the thickness of each layer is given in nanometers in the subscript. The Cu layer (on top of the Co layer) is used to break the Dzyaloshinskii-Moriya interaction as explained in Jhuria *et al*. (*23*). We then fabricated a CPS microwave waveguide structure with the magnetic devices embedded in series with both conductor lines using ultraviolet lithography and electron-beam evaporation. The CPS consists of two Ti(20 nm)/Au(300 nm) electrode lines which connect the Auston switch with the two magnetic samples, such that the current flow is along opposite directions through these two lines. Details about the sample fabrication can be found in (*21*, *23*).

### Time-resolved magnetization dynamics measurement

We have generated the ~9-ps current pulse by focusing a ~100-fs-duration laser pulse centered at 800-nm wavelength, with 0.28 μJ of energy from a 252-kHz regeneratively amplified Ti:sapphire laser on the ±50 V–biased Auston switch (with interdigitated electrodes and an overall area of 100 × 75 μm^2^). A weaker, synchronized 800-nm wavelength pulse with 0.13 nJ of energy, called the probe, was focused through a 50×, long working distance microscope objective (from Mitutoyo) to ~1.6-μm diameter on the magnetic sample. The magnetization dynamics were measured in a stroboscopic fashion, by varying the relative time delay between the pump and probe pulses using a motorized delay stage and time-averaging the MOKE signal for each delay. We perform the time-resolved magnetization switching experiments with an out-of-plane bias magnetic field (*H_z_*) (for details, see section S1) which exceeds the coercivity of the sample to restore the ferromagnet to the same saturated initial state before each repetition of the ~9-ps current pulse. The Auston switch and the magnetic elements are separated by more than 2 mm to fully avoid any unwanted direct optical effects in the magnetization dynamics.

### Current and energy density estimation

The FWHM of the current pulse sets an upper limit of the *RC* constant of the Auston switch (*RC* < 9 ps) and we can estimate a maximum capacitance (*C*_max_) of ~12.8 × 10^−14^ F (as the CPS impedance is ~70 ohms). This translates to a maximum of ~160 pJ of electrical energy $\left(E_{\max}=\frac{1}{2}C_{\max}V^{2}\right)$ stored in the Auston switch. Assuming that all this energy is transferred to the ~9-ps Gaussian current pulse and ultimately delivered to the magnetic microdot (~5 × 4 μm^2^), a maximum electrical energy density of ~0.81 mJ/cm^2^ could be delivered to the sample. The maximum current density (*J*_*c*,max_) that can be inferred from the electrical energy is ~9.6 × 10^12^ A/m^2^ [*E*_max_ = ∫ $J_{c,\max}^{2}$(*t*)ρ*dt*]. The actual current density in the device will be smaller than this value due to various losses, which is consistent with the current density (7.3 × 10^12^ A/m^2^) obtained in the macrospin simulations.

### Microscopic simulation

We performed microscopic spin simulations with UBERMAG (*34*) Object Oriented Micromagnetic Framework (OOMMF-based simulation technique in Python framework) due to a current pulse with 9-ps FWHM duration (although at 0 K and without including any ultrafast heating). The details can be found in section S10. In the absence of τ_TAT_, we do not obtain magnetization switching with a current density of 7.3 × 10^12^ A/m^2^. The minimum current density required to obtain switching is 16.5 × 10^12^ A/m^2^, when the magnetization drops close to zero within the full duration of the current pulse and it crosses the equator plane within ~40 ps. We do not observe any domain wall nucleation within this time frame and the switching occurs via a coherent rotation of the magnetization of the whole sample without any noticeable incubation delay. The microscopic simulation emphasizes the need for τ_TAT_ in obtaining ultrafast switching at a smaller current density and establishes the coherent switching mechanism even in the microspin picture with approximately picosecond current pulses. However, when we perform the simulation via exciting the magnet with a 1-ns square wave electrical pulse, we do observe domain wall–driven magnetization switching at a smaller current density. Our micromagnetic simulations estimated a much smaller switching current density when using a sub-100-ps current pulse, while maintaining the coherence of the magnetization switching and an ultrafast magnetization zero crossing. Details of the microscopic simulation can be found in section S10. For details about the macrospin simulation (which includes the ultrafast heating effect and is used to explain the experimentally measured magnetization dynamics), see section S4.

## Supplementary Materials

### This PDF file includes:

Sections S1 to S10 Figs. S1 to S12 Table S1
