# Supplementary material for: Picosecond spin-orbit torque–induced coherent magnetization switching in a ferromagnet
Source: Sci Adv. 2023 Sep 6;9(36):eadh5562. doi: 10.1126/sciadv.adh5562 (PMC10482325; doi:10.1126/sciadv.adh5562)
Supplement: Supplementary file 1 — Sections S1 to S10 Figs. S1 to S12 Table S1 [file sciadv.adh5562_sm.pdf]

Science Advances
AAAS

Supplementary Materials for

Picosecond spin-orbit torque–induced coherent magnetization switching in a ferromagnet

Debanjan Polley *et al.*

Corresponding author: Debanjan Polley, d.polley@berkeley.edu; debanjap@srmist.edu.in

Sci. Adv. **9**, eadh5562 (2023)
DOI: 10.1126/sciadv.adh5562

This PDF file includes:

Sections S1 to S10
Figs. S1 to S12
Table S1

Section 1: Hysteresis loops in the presence of an in-plane magnetic field

Due to the proximity of the in-plane (H_x) and out-of-plane (H_z) external magnets in our experimental setup (as shown in Fig. S1a), the in-plane magnet provides a prominent out-of-plane magnetic field as the field lines bend towards the core of the out-of-plane magnet.

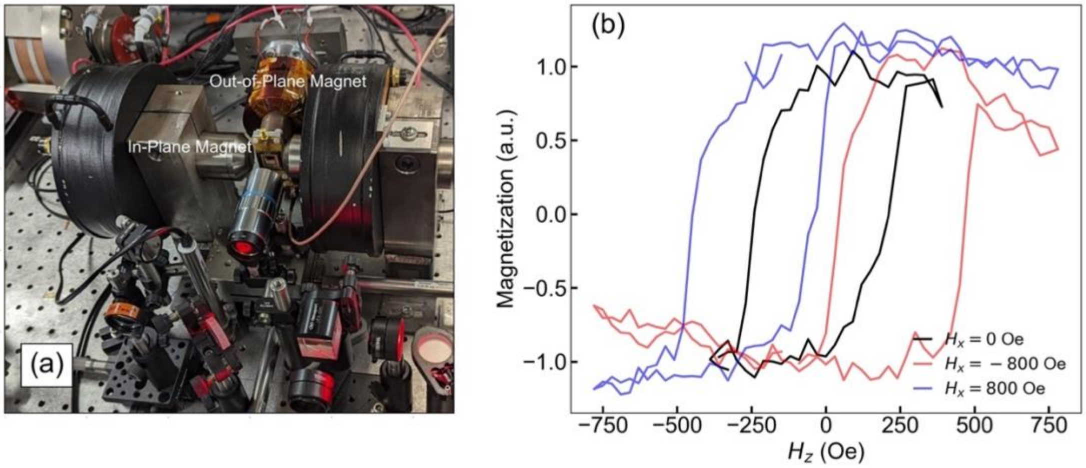

Fig. S1. Magnet setup and hysteresis loop. (a) The proximity of in-plane and out-of-plane magnets and (b) the out-of-plane hysteresis loops in the presence of different in-plane magnetic fields.

This effect can be observed from the shifts in the hysteresis loops of the Co microdot in the presence of 0 and $\pm$ 800 Oe in-plane field as shown in Fig. S1b, where the out-of-plane magnetic field is plotted in the x-axis. As we observe from this figure, even an 800 Oe in-plane magnetic field has almost enough out-of-plane components to switch the magnet. Hence, during time-resolved measurement with 1600 Oe in-plane field, we haven't applied any out-of-plane magnet separately and used the out-of-plane component of the in-plane magnet to provide an out-of-plane saturation magnetic field. However, for the time-resolved measurements with an 800 Oe in-plane magnetic field, we have explicitly used the out-of-plane magnet to provide the out-of-plane saturation field. In all cases, a saturating out-of-plane field is required in the repetitive pump-probe time-resolved measurements to reset the magnet back to the same initial condition for each successive cycle.

Section 2: Characteristics of the Auston switch; current-pulse width and ultrafast temperature increment dependence of the ultrafast switching

The IV characteristics of the photoconductive (Auston) switch are shown in Fig. S2a. We use a $60\ \mathrm{mW}$ pump power (at $252\ \mathrm{KHz}$ repetition rate) to study the photo-current and the measured photocurrent is ~ 10 times larger than the dark-current at the largest bias voltage (i.e. $50\ \mathrm{V}$). We highlight that the Auston switch gets saturated with $60\ \mathrm{mW}$ pump power (at $252\ \mathrm{KHz}$ repetition rate; $0.28\ \mu\mathrm{J}$) and at a fixed voltage, the photocurrent doesn't increase much beyond this pump power. We use a THz probe tip from Protemics GmbH on top of the co-planar strip-line (CPS), which measures the THz field generated from the THz current pulse propagating through the CPS. The measured FWHM of the current pulse width is ~ 9 ps.

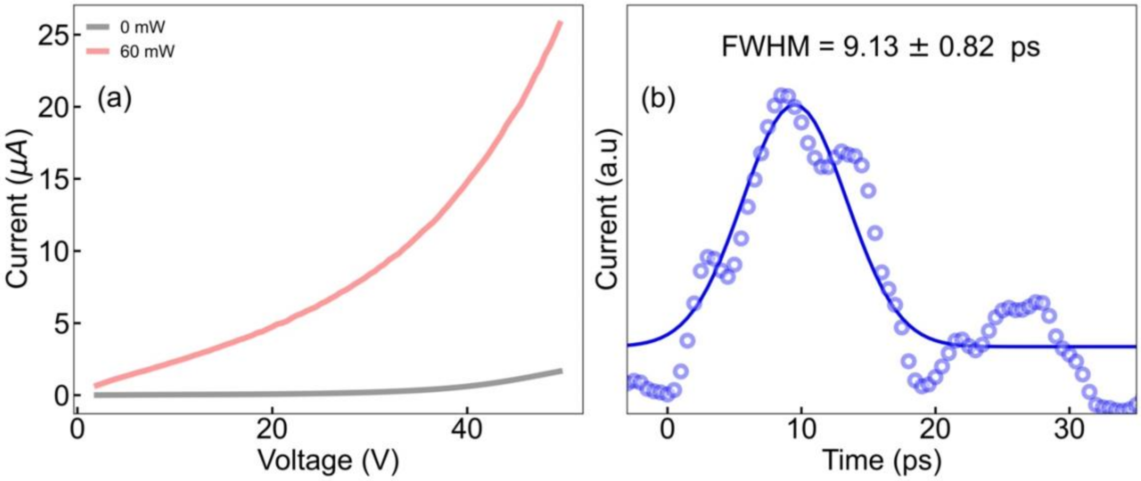

Fig. S2. Auston switch characterization. (a) The dark-current (black) and photo-current (red) as a function of the applied bias voltage and (b) the measured current pulse from the Auston switch using a THz probe tip from Protemics GmbH is shown in blue dots. The solid blue line is a Gaussian fitting showing ~9 ps FWHM.

The FWHM of the current pulse depends on the Auston switch configuration, waveguide structure, and the LT-GaAs substrate. We generally observe a current pulse width variation between $\sim 6 - 10$ ps using the amplified laser excitation in different types of Auston switches. As expected, one would require a larger current density for the smaller current pulse width to observe switching and vice-versa [13]. Using our microscopic simulation (details of which are given in section 4), we have calculated that with a 6 ps and 15 ps current pulse-width, we need $7.5 \times 10^{12}\ \mathrm{A/m^2}$ ($5.5 \times 10^{12}\ \mathrm{A/m^2}$) current density to observe a similar ultrafast SOT-induced magnetization switching which is shown as shown in Fig. S3. We use an in-plane symmetry-breaking field of $1600\ \mathrm{Oe}$. The main takeaway is that the physics of the switching remains the same within a small variation in the current pulse width. The switching mechanism is still

dominated by coherent rotation of magnetization without any noticeable incubation delay.

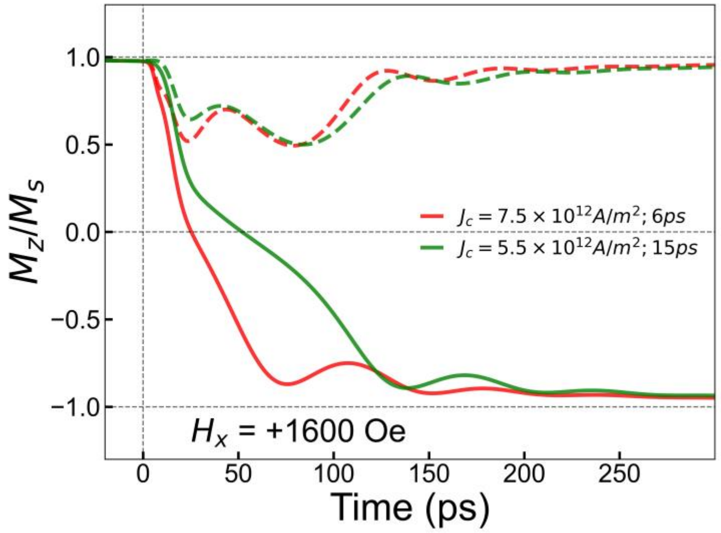

Fig. S3. Simulated effects of current pulse-width. The simulated magnetization dynamics using a 6 ps and 15 ps current pulse that requires a current density of 7.5×10^{12} A/m^2 and 5.5×10^{12} A/m^2 respectively to induce ultrafast SOT induced magnetization switching. The solid (dotted) lines are obtained using a positive (negative) current pulse in the presence of 1600 Oe in-plane symmetry-breaking magnetic field.

To gain some insight into the reduction in critical current due to ultrafast heating, we have carried out the macroscopic simulation with and without the ultrafast thermal effect for the same ~9 ps current pulse as shown below in Fig. S4. We observe that the magnetization doesn't switch with the same current density (7.3×10^{12} A/m^2) if we don't include the ultrafast thermal heating effects. The magnetization can be switched even if we don't include the ultrafast heating effect but require a much larger current density (13.8×10^{12} A/m^2) as shown in Fig. S4b.

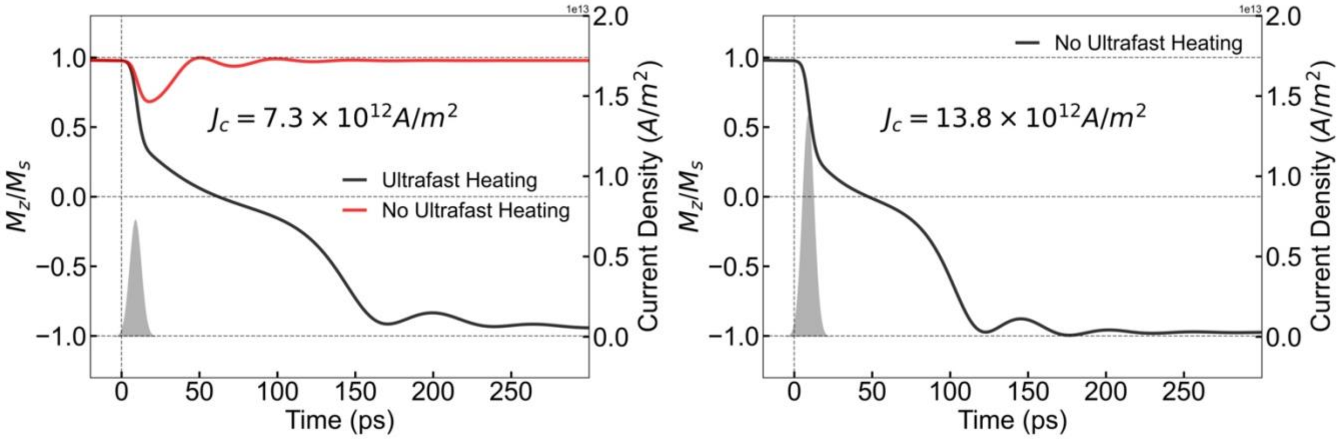

Fig. S4. Simulated effects of ultrafast heating. The simulated magnetization dynamics due to (a) a current density of 7.3 $\times 10^{12}$ A/m^2 with and without ultrafast heating effect and due to (b) a current density of 13.8 $\times 10^{12}$ A/m^2 without ultrafast heating effect. The red Gaussian pulse shows the 9 ps current pulse with respective current densities.

Section 3: Single-shot MOKE switching images

In the presence of 1600 Oe in-plane magnetic field, we acquire the first magneto-optical Kerr effect (MOKE) image after saturating the sample along H_{+z} or H_{-z} (i.e. its magnetization starts from M_{+z} or M_{-z} orientation, respectively) and then capture the second MOKE image after passing a single ~ 9 ps current pulse (SS) through the electrodes (by single-shot optical excitation of the Auston switch under a 50 V bias). The sign of the $\sim$ps current pulses along the two electrodes is shown by the green arrows at the top and bottom rows of the schematic for positive and negative biases, respectively, across the Auston switch. The difference between these two images is shown in the first and second columns of Fig. S5 respectively. The black (or white) contrast signifies a complete switching of the magnet from M_{+z} to M_{-z} (or M_{-z} to M_{+z}) magnetic saturation state and the grey contrast signifies no change in the magnetic state. In the top-left quadrant of Fig. S3, the top magnetic micro-dot switches, which means it prefers M_{-z} orientation (shown by the black contrast) for the parallel combination of in-plane magnetic field and $\sim$ps current pulse directions. However, the bottom microdot doesn't switch (shown by the grey color). Similarly, if we start the experiment from M_{-z} orientation, the top magnetic micro-dot doesn't switch, and the bottom microdot switches to M_{+z} as shown by the white contrast. A similar switching phenomenon is observed by reversing the bias voltage of the Auston switch (bottom two micrographs of Fig S5). In this case, the final magnetization directions are opposite to the final states of the top two micrographs since the current directions have been reversed. These MOKE contrasts indicate that the final magnetic states of the microdot are independent of the initial states and depend only on the relative orientation of the current pulse and in-plane magnetic field as expected from the symmetries of the SOT in the magnetic heterostructures [13, 23]. We repeated each measurement five times and observed the same final magnetic switched state. Lowering the bias voltage below 50 V, without increasing the in-plane magnetic field, results in no switching.

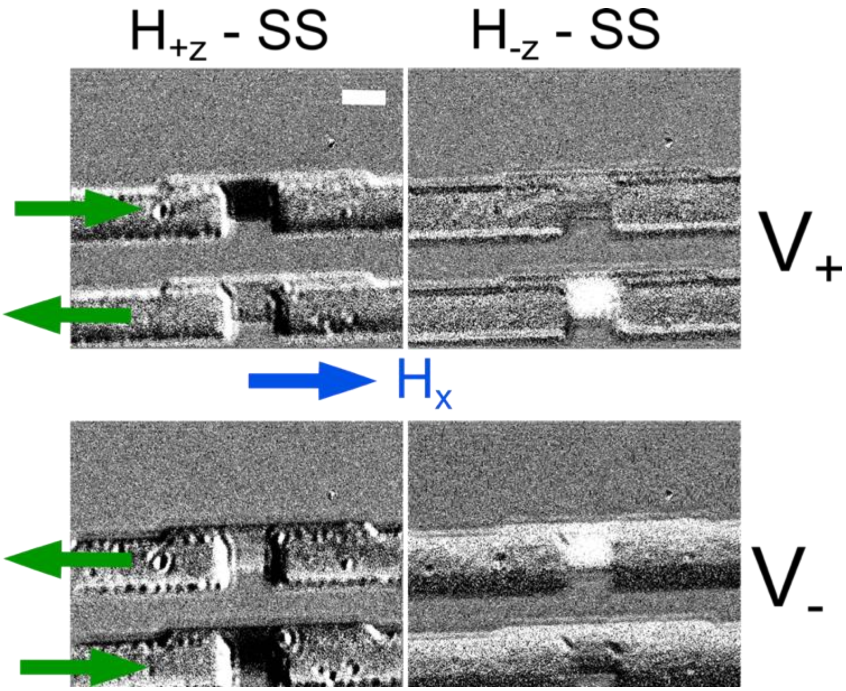

Fig. S5. Single-shot MOKE imaging. The differential magneto-optical Kerr effect (MOKE) images showing the reversal of the magnetization due to a single current pulse (green arrow) in the presence of 1600 Oe in-plane symmetry breaking magnetic field (H_x), under positive and negative bias voltages (V_+ and V_-) across the Auston switch, starting from a positive and negative magnetic saturation. We capture two MOKE images; 1) after saturating the magnets either along H_{+z} or H_{-z} direction and ii) after passing a single ~9 ps current pulse (SS) and the subtraction of these two images produces the differential MOKE contrast, where if a switching event occurs from H_{+z} to H_{-z} (or H_{-z} to H_{+z}), it appears as black (or white) contrast, and a gray represents no change in magnetization. The scalebar is 5 µm.

Section 4: LLG Simulation with ultrafast heating

We have adopted the same technique used in one of our previous works to study SOT-induced magnetization dynamics [23]. The LLG differential equation for a ferromagnet in an external field can be extended by linearity to include the effect of the spin current as given below.

$$\frac{dM(t)}{dt} = -\gamma\mu_0\left(\vec{M} \times \overrightarrow{H_{eff}}\right) + \frac{\alpha}{M_s(T)}\left(\vec{M} \times \frac{dM(t)}{dt}\right) + \Theta_{FL}C_s\left(\vec{M} \times \vec{\sigma}\right) - \Theta_{DL}\frac{C_s}{M_s(T)}\left(\vec{M} \times \vec{M} \times \vec{\sigma}\right).$$

Here, γ, μ_0, H_{eff}, α, and σ are the gyromagnetic ratio, vacuum permeability, effective magnetic field, Gilbert damping, and spin-polarization direction, respectively. The torques on the magnetization due to the spin current's magnetic moment is modulated by the field-like spin Hall angle and the damping spin Hall angle. In the first two terms detailing the interaction of the ferromagnet with the external field, μ_0 is the permeability of free space, γ is the gyromagnetic ratio of electrons, $\vec{M}$ is the magnetization of Cobalt,

H_{eff} is the effective external field which consists of the external field, demagnetization field, and magneto-crystalline anisotropy, α is Gilbert damping parameter, and M_s is the saturation magnetization. In the last two terms that incorporate the action of the spin currents, Θ_{FL} and is the field-like and anti-damping spin Hall angles, respectively, and σ is the unit vector that points in the same direction as the spin current's magnetic moment while the magnitude of the torque's effect is absorbed into the C_S variable, where, $C_S = \frac{\mu_B J_c}{q_e d_0 M_s}$, and μ_B, J_c, q_e and d_0 are the Bohr magnetron, time-varying current density, the elementary charge of an electron and the thickness of Cobalt respectively. After solving the LLG equation, we obtain m_x, m_y and m_z in reduced units (the norm of the magnetization still 1). We multiply m_z with $\left(M_s(T) / M_s(300\ K) \right)$ to obtain the normalized out-of-plane magnetization $\left(M_z / M_s = m_z \times \left(\frac{M_s(T)}{M_s(300\ K)} \right) \right)$ as shown in the main text. As the magnetic sample has PMA, we assume the ultrafast demagnetization mostly affects the z component, hence the in-plane magnetization components are normalized as $M_x / M_s = m_x \times \left(\frac{1}{M_s(300\ K)} \right)$ and $M_y / M_s = m_y \times \left(\frac{1}{M_s(300\ K)} \right)$.

Now, as we already know, with ~ps current injection in a metallic magnetic stack, the thin films undergo non-equilibrium Joule heating, and with the peak electron temperature rising much higher than room temperature and thereby it shows ultrafast demagnetization [21, 23]. Hence, we have considered the thermal evolution of the saturation magnetization and the anisotropy in the LLG simulation. If the metallic stack is assumed to be symmetric in two dimensions and varies only in one direction, the temperature response of the metal film can be estimated by solving the following one spatial dimension heat-diffusion equation.

$$C \frac{\partial T(x,t)}{\partial t} = \Lambda \frac{\partial^2 T(x,t)}{\partial x^2} + q(t).$$

Here, $T(x,t)$ is the temperature of the film, C is the volumetric heat capacity, Λ is the metal's thermal conductivity, and $q(t)$ is the external power per unit volume generated due to the electric pulse. Assuming Ohmic behavior, volumetric heating due to the current can be estimated using $q(t) = \rho_e J^2(t)$, where $\rho_e = 10\ \Omega nm$ is the measured resistivity across the stack and $J(t)$, is the current density. The volumetric heat capacity of the full metallic stack is assumed to be, $C = 1.75\ \mathrm{Jm^{-3}K^{-1}}$, and the thermal conductivity of the stack is taken to be 9 $\mathrm{Wm^{-1}K^{-1}}$. We have used here a one-temperature model as we know the electron-lattice thermalization timescales in these materials are in the range of several ~ps, when the system can effectively be described by a single temperature without introducing crucial error. The evolution of

saturation magnetization and anisotropy is given by the following equation (and shown in Fig. S6):

$$M_s(T) = M_s(0\ K)\left[1 - \frac{T}{T_c}\right]^{1.7}$$

$$K_z(T) = K_z(0K)\left[{M_s(T)}\big/{M_s(0\ K)}\right]^{3.0}.$$

Here, T_c is the Curie temperature of Cobalt, $M_s(0\ K)$ and $K_z(0\ K)$ is the saturation magnetization at anisotropy constant at absolute zero. The value of $M_s(T = 300\ K)$ is fixed to $10^6\ \mathrm{Am^{-1}}$ from magnetometry measurements.

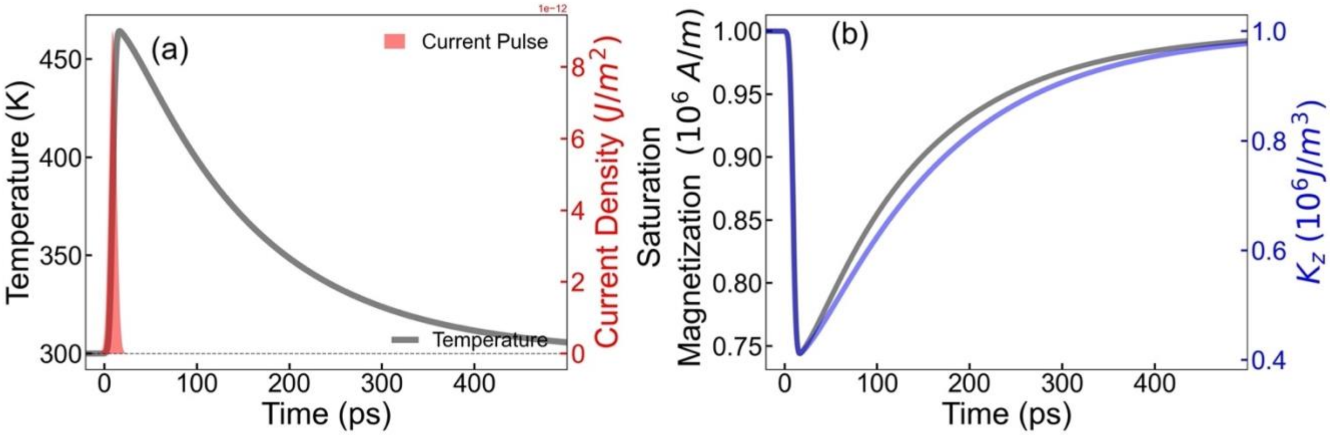

Fig S6. Simulated effects of the ultrafast temperature change on magnetic parameters. (a) Time-resolved temperature dependence due to the application of the ps current pulse depicted by the red filled region and (b) the time-resolved variation of the Magnetization (black line) and Uniaxial anisotropy (blue line).

The parameters used in this simulation are given in the following table.

Parameter	Value
Volumetric Heat Capacity (C)	$1.75 \times 10^{6}\ Jm^{-3}K^{-1}$
Interfacial Thermal Conductance (G)	$200 \times 10^{6}\ Wm^{-2}K^{-1}$
Thickness of the Magnetic Layer (d_0)	$1\ nm$
Total Thickness (d)	$16\ nm$
Thermal Conductivity of the Metal (Λ)	$9\ Wm^{-1}K^{-1}$
Electrical Resistivity (ρ_e)	$1.0 \times 10^{8}\ \Omega m$
Current Density (J_c)	$7.3 \times 10^{12}\ Am^{-2}$
FWHM of the Current Pulse	$9\ ps$
Saturation Magnetization (M_s)	$10^{6}\ Am^{-1}$
Anisotropy Constant (K_z)	$10^{6}\ Jm^{-3}$
Gilbert Damping (α)	0.22
Curie Temperature (T_c)	$800\ K$
Out-of-plane Magnetic Field (H_z)	$300\ Oe$
Symmetry-Breaking In-Plane Magnetic Field (H_{in})	$1600\ Oe$

Table. S1. Parameters used in the macro-spin simulation. Parameters used in the macroscopic LLG simulation with ultrafast heating.

Section 5: Ultrafast optical demagnetization

We use an incident optical pump fluence of $\sim 3.7\ \mathrm{mJ/cm^2}$ to study the ultrafast optical demagnetization on a thin film of the sample as shown in the blue circles in Fig. S7. From the transfer function calculation, we found $\sim 8\ \%$ of the incident fluence ($\sim 0.3\ \mathrm{mJ/cm^2}$) is being absorbed by the 1 nm thick Co layer. We experimentally observed an ultrafast thermal demagnetization of $\sim 50\ \%$. The maximum electron temperature rise is much larger due to the ~fs nature of the excitation (compared to a ~ps excitation) causing stronger demagnetization. We have used the following formulae to fit the demagnetization curve:

$$\frac{\Delta M_Z(t)}{M_S} = \left[\frac{A_1\tau_e - A_2\tau_m}{\tau_e - \tau_m} e^{-t/\tau_m} - \frac{\tau_m(A_1 - A_2)}{\tau_e - \tau_m} e^{-t/\tau_e}\right] \otimes [G(t)],$$

where τ_m and τ_e are the ultrafast demagnetization and fast remagnetization time, A_1 and A_2 are related to ultrafast demagnetization amplitude, and $G(t)$ is the Gaussian pump pulse with 100 fs FWHM for optical and electrical excitation $\otimes$ denote a convolution between the two elements. We extract a 115 ± 38 fs demagnetization time and a remagnetization time of 1.34 ± 0.16 ps for optical excitation with the corresponding fitting shown as the dotted blue line in Fig. S7 using the above-mentioned model.

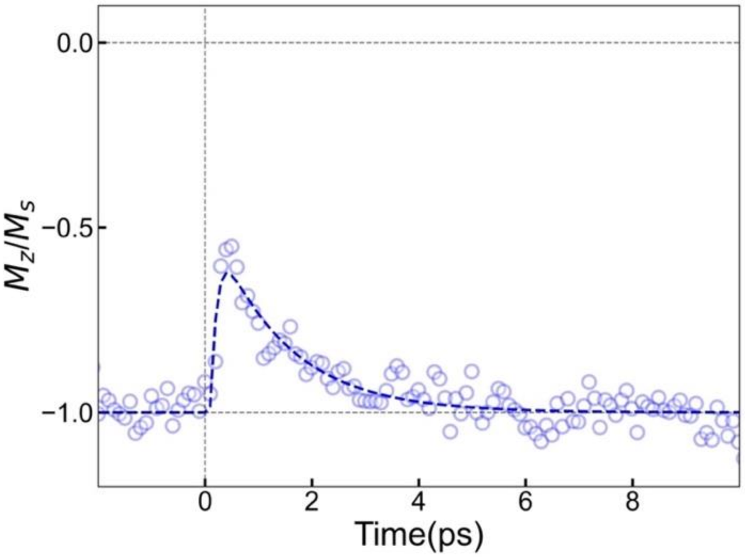

Fig S7. Ultrafast optical demagnetization. Ultrafast Optical Demagnetization due to ~100 fs optical pulse as shown by the blue circles and the corresponding fitting with an empirical formula discussed in the text is given by the solid blue line.

We note that the remagnetization time is much faster and doesn't go in line with the electrical magnetization measurement. We don't completely understand this but might be due to the different heat diffusion and slightly different magnetic properties between the ferromagnetic thin film (where optical demagnetization is measured) are measured and the ferromagnetic microdot (where electrical magnetization dynamics were studied).

Section S6: Long time-delay measurements

The long time-delay scans of the time-resolved magnetization dynamics show that the magnetization doesn't recover completely after the current pulse excitation (measured till ~600 ps) as shown in Fig. S8. This suggests that the current pulse-induced thermal heating remains in the systems over this time. However, using the one-dimensional heating effect introduced in our macroscopic simulation model, the effect of the heating is not correctly reproduced, maybe the effect of individual interfaces along the multilayers structure and its diffusion through the MgO and LT-GaAs layer is required to match the experimentally observed long-time delay magnetization profile.

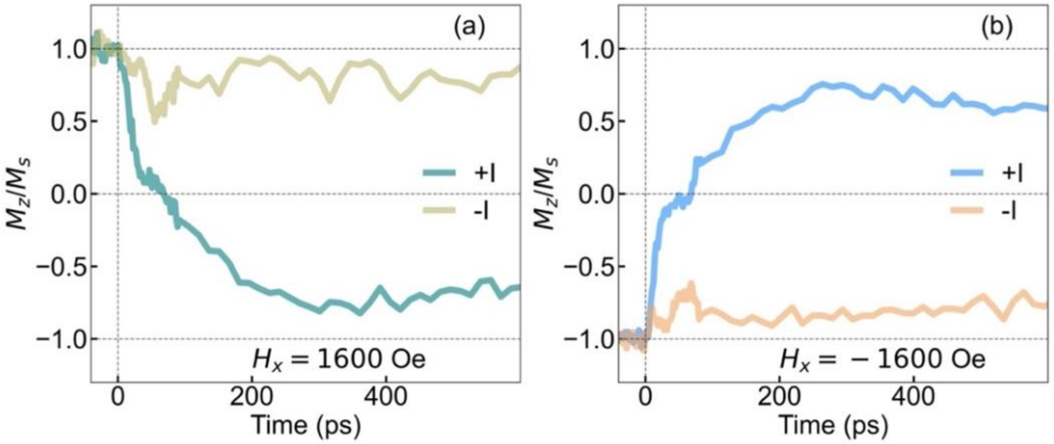

Fig. S8. Long time-delay scan. The ∼9 ps electrical pulse-induced time-resolved magnetization dynamics were measured in the presence of (a) positive and (b) negative in-plane symmetry breaking magnetic field and reversing the direction of the current pulses starting from positive and negative magnetic saturation. The solid (faint) lines denote the magnetization dynamics using positive (negative) ∼9 ps current pulse.

.

Section S7: The effect fluence on the Auston switch

We have used different fluence (0.28, 0.30 and 0.32 μJ) on the Auston switch at a fixed bias voltage of ± 50 V and studied the magnetization dynamics as shown in Fig. S9 in the presence of a 1600 Oe in-plane symmetry breaking magnetic field. The zero-crossing time and the overall shape of the magnetization dynamics don't change with increasing fluence on the Auston switch by ∼20 %. This further strengthens our claim that we are operating the Auston switch at its saturation with 0.28 μJ pump energy on the Auston switch. Hence, the calculation of the capacitance of the Auston switch from the FWHM of the current pulse and the corresponding maximum energy stored in the Auston switch is valid throughout the magnetization switching experiments.

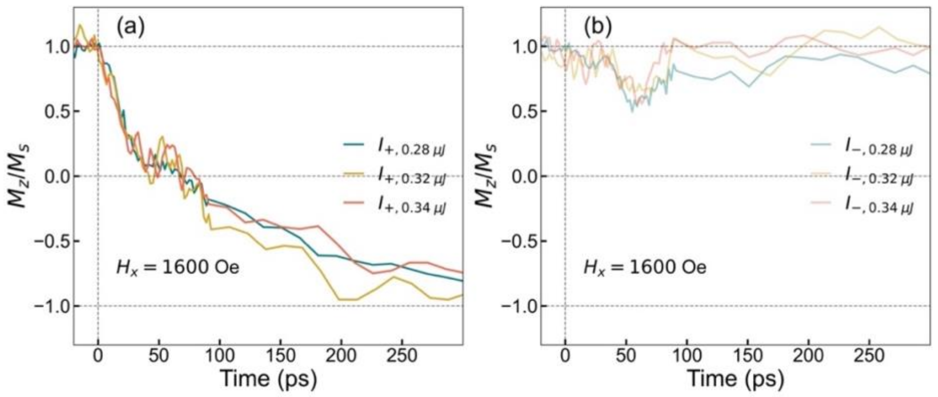

Fig. S9. Effects of Fluence. The magnetization dynamics in the presence of 1600 Oe in-plane magnetic field for (a) positive and (b) negative ~9 ps current starting from a positive magnetic saturation for different pump energy on the Auston switch.

Section S8: The effect of in-plane magnetic field

It is well-known that a symmetry-breaking in-plane magnetic field is required for SOT-induced switching in perpendicular magnetic anisotropy magnets [12, 23]. In our case, we need ~1600 Oe in-plane magnetic field to observe switching as shown in the single-shot MOKE micrographs in Fig. S5 in the earlier section. Magnetization switching isn't observed when we used a smaller field, as shown in Fig. S10a. With a lower in-plane field, a larger current density is likely needed to initiate switching. The time-resolved magnetization spectra are shown in Fig. S10b for both positive and negative current pulse direction while the dashed lines show the simulated results. The macroscopic simulation shows similar partial demagnetization behavior for a ~9 ps current pulse with a current density of $7.3 \times 10^{12} \mathrm{A/m^2}$.

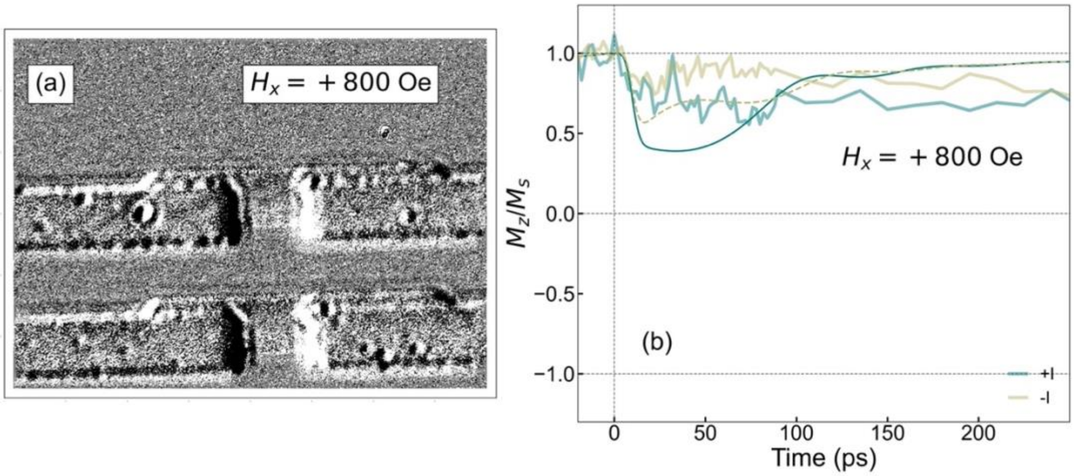

Fig. S10. Effect of the in-plane magnetic field. (a) The single-shot MOKE micrograph starting from positive magnetic saturation in the presence of 800 Oe in-plane symmetry-breaking field. The contrast in the magnetic sample doesn't change upon single-shot current pulse excitation which demonstrates no magnetization switching. (b) The corresponding magnetization dynamics are demonstrated. The simulated spectra shown in dotted lines for positive (green) and negative (yellow) current pulses show reasonable agreement with the experiment.

Section 9: Effect of the field-like torque

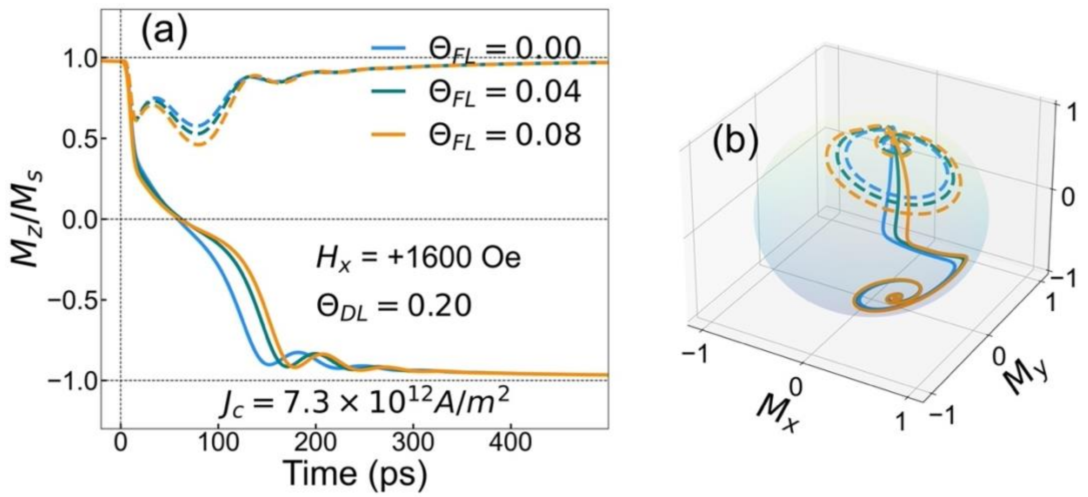

Fig. S11. Simulated effects of the field-like torque. Time-resolved magnetization dynamics in the presence of 1600 Oe in-plane magnetic field due to a ~9 ps current pulse with a current density of 7.3×10^{12} A/m^{2}for positive (solid) and negative (dotted) direction at three different field-like torque angles and the corresponding (b) three-dimensional magnetization profile.

We have shown the effect of the field-like torque angles while keeping the damping-like torque ($\Theta_{DL} = 0.2$) constant at a fixed current density ($J_c = 7.3 \times 10^{12}$A/m^2) of the 9 ps current pulse in the presence of a positive 1600 Oe in-plane symmetry breaking magnetic field for both positive (solid) and negative (dotted) current directions. The field-like torque increases the amplitude of the oscillation during remagnetization as shown in the dotted lines in Fig. S11a. During magnetization switching, the magnetization crosses zero ~70 ps and it doesn't change with increasing Θ_{FL}. However, magnetization settles to the negative saturation value, much faster without any field-like torque (black solid line). The negative saturation gets delayed with increasing field-like torques within our simulated range.

Section S10: Switching mechanism via micro-magnetic simulation

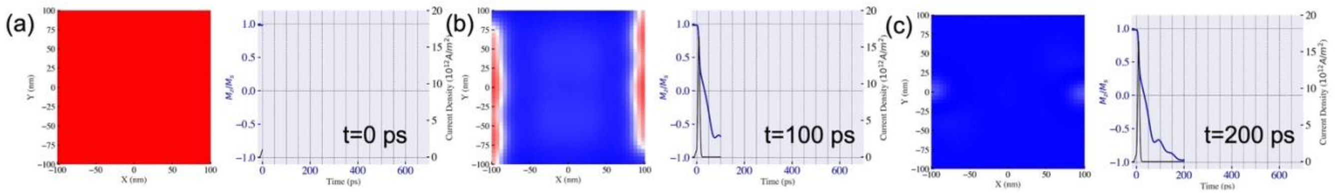

Fig. S12. Micro-spin simulation. (a-c) UBERMAG based micromagnetic simulation shows the coherent rotation of magnetization due to the application of the 9 ps Gaussian current pulse with a current density of 16.5×10^{12} A/m^2. The color map shows the microscopic magnetization dynamics (red means M_{+z} and blue means M_{-z}) at different timescale, the corresponding current profile and the normalized magnetization along z-axis is shown in the time-resolved graph.

We have used UBERMG [34] to microscopically simulate the magnetization dynamics using a simple model (at 0 K and without introducing any ultrafast heating-related demagnetization) using damping-like and field-like terms in the LLG equation and exchange, demagnetization, and uniaxial anisotropy energy in calculating the total energy of the system. We need to increase the current density to $16.5\ \times 10^{12}\ \mathrm{A/m^2}$ to observe switching as shown in Fig. S12 a-c. Without the ultrafast thermal anisotropy torque, we do not observe magnetization switching for a current density with $7.3\ \times 10^{12}\ \mathrm{A/m^2}$. This establishes the need for thermal anisotropy torque in observing the ultrafast switching with a smaller current density. We observe an ultrafast switching with zero crossing at ~ 40 ps and complete switching in ~ 150 ps. We notice that a coherent rotation of the magnetization is the responsible mechanism for the switching phenomenon (even without the presence of any ultrafast demagnetization). We used a magnetic sample size of $100\ \mathrm{nm} \times 100\ \mathrm{nm} \times 1\ \mathrm{nm}$ and the discretized cell size is $5\ \mathrm{nm} \times 5\ \mathrm{nm} \times 1\ \mathrm{nm}$, hence basically it is a 2d model with no discretization along the z-axis. A ~ 9 ps Gaussian current pulse with a current density of $16.5\ \times 10^{12}\ \mathrm{A/m^2}$ and a $\Theta_{DL} = 0.2$ and $\Theta_{FL} = 0.04$; is used in the simulation where the other magnetic parameters (Saturation magnetization, Uniaxial anisotropy, and Damping Constant) are the same as used in the Macroscopic simulation model discussed in detail earlier and in the main manuscript. The current pulse is plotted along the right Y axis and the magnetization is plotted along the left Y axis. Magnetization of the sample turned from red (M_{+z}) to blue (M_{-z}) via a coherent rotation with time as a function of the current pulse propagation. We don't observe any formation of domain walls in the magnet as evident from the magnetization images. However, when using a 1 ns current pulse (not shown here), we do observe the formation of the domain wall at the center of the sample and its propagation along the edges suggesting a domain-wall driven switching mechanism for ~ns long current pulse.
